# From polarity to plurality: Perceptions of COVID‐19 and policy measures in England and Scotland

**DOI:** 10.1111/hex.14069

**Published:** 2024-05-11

**Authors:** Jack Rendall, Neil McHugh, Rachel Baker, Helen Mason, Olga Biosca

**Affiliations:** ^1^ The Yunus Centre for Social Business and Health Glasgow Caledonian University Glasgow UK

**Keywords:** COVID‐19, plurality, polarisation, public perceptions, Q methodology

## Abstract

**Aim:**

The aim of this study was to uncover perspectives on the COVID‐19 pandemic and the responses implemented by the UK and Scottish Governments to help control the spread of infection. Such understanding could help to inform future responses to pandemics at individual, community and national levels.

**Method:**

Q methodology was used to elicit perspectives from people in England and Scotland with different experiences of the pandemic including public health officials, key workers, those on furlough, those who were unvaccinated or vaccinated to different levels, those who were ‘shielding’ because they were at higher risk and people with different scientific expertise. Participants rank‐ordered phrases about different aspects of COVID‐19 according to their viewpoint. Factor analysis was then conducted in conjunction with interview material from the same respondents.

**Results:**

A four‐factor solution was statistically supported and was interpretable alongside the qualitative accounts of participants loading on these factors. These four perspectives are titled Dangerous and Unaccountable Leadership, Fear and Anger at Policy and Public responses, Governing Through a Crisis and Injustices Exposed.

**Conclusion:**

The four perspectives demonstrate plurality and nuance in views on COVID‐19 and the associated policies and restrictions, going beyond a binary narrative that has been apparent in popular and social media. The four perspectives include some areas of common ground, as well as disagreement. We argue that understanding the detail of different perspectives might be used to build cohesion around policy initiatives in future.

**Patient or Public Contribution:**

The development of the statement set, which is rank‐ordered by participants in a Q study, and factor interpretations were informed by views of the general public. The statement set was initially developed using existing publicly available material based on members of the general public experiencing the pandemic first hand. It was then piloted with members of the public experiencing different challenges as a result of COVID‐19 and the subsequent lockdown and updated based on feedback. Finally, interpretations of the identified factors were presented publicly and edited according to their feedback.

## 1 INTRODUCTION

Over 6.9 million deaths to date have been attributed to COVID‐19 globally, with over 230,000 of those in the United Kingdom, at the time of writing.^1^ Confronted with such a crisis, different views on the best course of action to protect ourselves, our loved ones, our communities and our society at large proliferated the public consciousness. This crisis led to communities pulling together to help one another.^2^ Paradoxically, COVID‐19 also provided fertile ground for polarisation. At the same time as reports of remarkable solidarity and kindness in the United Kingdom, conflicting perspectives and polarisation of public views led to a ‘multiplicity of differences’ and a state of ‘Us vs Them’.^3^ ^,p.16^ Such polarisation stemmed from a ‘climate of fear, insecurity, and competition for scarce resources’^4^ ^,p.605^ and has been evident in the media, in political discourse and in everyday conversations, online and offline. Polarised responses to public health emergencies are not new. The Spanish flu pandemic of 1918 has been much cited during the COVID‐19 pandemic. Many public health edicts were the same then as now—that people should avoid meeting indoors, or gathering in large groups, mask wearing became mandatory in many places^5^ and history documents protests in response to those policy measures, including an ‘anti mask league’ in San Francisco.^6^

Despite evidence of polarisation, reducing the public narrative to one characterised by extremes such as, in the COVID pandemic, a depiction of ‘moral citizen’ versus ‘deviant conspiracist’ is likely to be unhelpful in future pandemics or public health emergencies. Kelly^7^ calls for a more nuanced approach to understanding the subjective experiences of different people in different contexts during COVID‐19 and ‘the multiple pathways of the origins and manifestations of the disease, of vulnerability, susceptibility and protection’.^7^ ^,p.20^ In this paper, we uncover the richness and plurality of perspectives. We report findings from a Q methodology study that explores perspectives on COVID‐19 and governments' policy responses to the pandemic in England and Scotland gathering data using a card‐sort technique and interview, between the period of October and December 2021. We describe four distinct perspectives and the similarities and differences between them.

### 1.1 Cohesion and polarity

High levels of solidarity in the form of social cohesion—the perceived ‘togetherness’ felt in society^8^—have been shown to have a positive impact on the ability of a community to recover from natural disasters at a faster rate.^9^ Communities' ability to recover from a shock such as a natural disaster has been described as stemming from their resilience.^10^ For the UK Government, resilience is a key area of interest in their COVID‐19 Public Enquiry (an independent enquiry into the UK's response and impact of the COVID‐19 pandemic, launched in July 2022). Societal or community resilience, it seems, depends on a high level of social cohesion. Evidence has shown that geographical areas with strong social cohesion were better able to cope with the various challenges of the COVID‐19 pandemic.^11^ There is also evidence that individuals with lower sense of social cohesion were associated with a lower antibody response to vaccination.^12^ Gallagher et al.^12^ suggest that this connection is mediated by a sense of loneliness, which can negatively impact upon the hormonal and immune system functioning with regard to the body's ability to fight infection.

In contrast with accounts of community solidarity such as the organising of community food parcels and prescription deliveries were increasingly polarised narratives about the virus and about the policy measures and restrictions to freedoms during the COVID‐19 pandemic.^13^ Research on communication during the pandemic (before the second wave) showed that information inequality was growing as the crises continued across the United Kingdom.^14^ Political polarisation more generally has, arguably, worsened in recent years in the United Kingdom^15^ alongside debates on the United Kingdom leaving the European Union, Scotland becoming an independent nation and immigration, and so perhaps public responses to the pandemic are ‘only’ a reflection of existing divisions. While solidarity and social cohesion had risen during the initial stages of the pandemic,^16^ this was followed by ‘subsequent increases in suspicion and decreases in interpersonal contact, together with potential declines in trust in governing institutions and in other people’, which suggested a lean towards ‘social disintegration rather than cohesion’.^11^ ^,p.541^

Like so many other social and health issues, the pandemic had an unequal impact on different groups of society,^17^, ^18^, ^19^, ^20^ not just in the United Kingdom but globally.^21^ Like the Spanish flu a century before, early claims that the ‘virus does not discriminate’ were overturned by evidence that infection rates and deaths were higher in more disadvantaged areas and in some ethnic groups. Indeed, academic researchers did not always agree on the best policy responses to COVID‐19. For example, the Scientific Advisory Group for Emergencies (SAGE) set up by the UK Government faced a ‘rebellious’ independent (iSAGE) group that sought to be transparent in their decision‐making, compared to the original SAGE group.^22^ Plurality in public viewpoints presents challenges for population health interventions and for public health messaging. Understanding plurality is crucial for developing effective crisis interventions and crafting targeted policies that can be implemented through improved public communication. This can potentially lead to increased uptake and adherence to such policies.

### 1.2 COVID‐19, public preferences and perspectives

There is a range of studies on public views and COVID‐19. We make a distinction here between two different types of studies: quantitative preference elicitation studies and qualitative studies on perspectives.

Preference studies are surveys that ask respondents to make trade‐offs between different choices, through which participants ‘state their preferences’. At various points during the pandemic, Discrete Choice Experiments have been designed, focussing on the health impacts of different COVID‐19 policies in the United Kingdom,^23^ features of lockdowns^24^, ^25^, ^26^ and social distancing strategies.^27^, ^28^ These generate important information, for example, the measures of harms or restrictions considered worthwhile to reduce infection risk. As with all research techniques, there are limitations; preference surveys necessarily simplify scenarios and choices and hence narrow the framing of the question.

Qualitative research has explored perspectives covering a variety of COVID‐19‐related issues. Some studies focus on particular aspects such as vaccines^29^ or antibody testing,^30^ whereas other studies looked at more general views of the pandemic.^31^, ^32^ These studies provide a depth that is hard to capture through preference studies because they can explore the reasons and rationales in detail. However, qualitative studies do not capture the relative importance that participants placed on, for example, trade‐offs between policy harms and benefits. Q methodology provides a road between qualitative data sets without prioritisation and choice‐based studies that lack qualitative richness and reasoning.

## 2 METHODS

Q methodology is a set of qualitative and quantitative techniques used to study subjectivity. Respondents rank order a set of stimuli (typically statements of opinion—see Table 1) onto a grid (see Appendix S1) according to instructions such as ‘most like my point of view’ to ‘most unlike my point of view’; this activity is called the card‐sort as typically the statements are printed onto small cards that are then sorted. Factor analysis is then used to help identify areas of similarity between card‐sorts and the resultant outputs are called factors. Each factor is represented by an idealised (or composite) card‐sort, which is a separate rank‐ordering of the original set of statements for each factor. The idealised card‐sort for each factor is a weighted average of the card‐sort of ‘defining’ participants (i.e., those with high, pure association with one factor over the others—see Table 2 flagged with ‘x’). The composite card‐sorts are interpreted alongside qualitative data drawn from interviews with, or written explanations by, participants after they have sorted their statements explaining their views and why they ranked cards very high or low on the grid. Q methodology was selected for this study because the combination of the quantitative factor analysis and the rich qualitative data from the semi‐structured interviews permits rich insights that traditional surveys and interviews could not achieve as two separate approaches. This methodology is widely used across a number of varied fields,^33^, ^34^, ^35^, ^36^ including the exploration of public perspectives on health.^37^ More details on the techniques and tools of Q methodology can be found elsewhere.^38^, ^39^

**Table 1:** Statements and ranks.

Statement number	Statements	Factor 1	Factor 2	Factor 3	Factor 4
1	This is just like a bad flu but they've dressed it up to be terrifying	−5	1	−5	−5
2	It's a tragedy that people are dying from preventable illnesses because of COVID‐19	2	6	5	2
3	COVID‐19 has changed our perceptions of risks to society	1	−2	4	2
4	We have become numb to the number of COVID‐19 deaths and infections	3	−2	−3	0
5	I believe that this pandemic made us realise that we need to adjust how we live and work	2	−1	5	5
6	We have learned lots of lessons from the pandemic that will make the world a better place	−2	−3	2	2
7	The COVID‐19 restrictions have been unfair	−3	5	−4	−1
8	*Predicting the time frame of the pandemic has been frustrating* a	0	0	0	1
9	The virus has really highlighted the over‐emphasis placed on the healthcare system relative to social programmes such as home care, care homes, social work	3	0	0	3
10	The health system was not strong enough to cope with the pandemic	6	1	−1	2
11	I am worried about variants of the virus	2	−3	1	−1
12	The government let down those who were already vulnerable like people living on low incomes, disabled people and those with pre‐existing health conditions	4	5	−2	2
13	What essential workers are paid should change to reflect how much we value them as a society	4	3	3	6
14	Systems to track those infected with the virus worked well	−3	−4	0	−4
15	People will always die. We need to learn to get used to living with COVID‐19 and keep things running	−3	2	−3	1
16	The long‐term effects of restrictions will be worse than the effects of COVID‐19 on individuals’ health	−1	4	−3	−1
17	Rich people are better able to protect themselves from the effects of the virus	0	2	0	6
18	The pandemic is having a devastating effect on people's mental health	6	6	4	3
19	Pretty much everyone is going to catch the virus in the end—it's just a matter of when	−2	3	2	4
20	The media have been responsible for causing paranoia and hysteria during the pandemic	0	5	3	0
21	Knowing there is a vaccine is a massive relief	4	−4	5	3
22	The pandemic has made us more aware of each other like never before	0	0	6	4
23	I've become paranoid about spreading the disease and others spreading it to me	1	−2	−1	−4
24	*To survive the pandemic businesses have been forced to innovate as otherwise they are being left to die*	0	0	2	0
25	A lot of the COVID‐19 impacts could have been avoided by shutting down sooner	5	−3	1	0
26	I have great belief that our government is doing everything in their power to keep people safe	−3	−3	4	−2
27	Individuals should be forced to follow government guidance	0	−4	−1	−3
28	I have been appalled at the behaviour of people during the pandemic	3	1	1	−4
29	*The pandemic shows the importance of having strong worker's unions* a	0	0	1	0
30	Business leaders have helped lead the fight against COVID‐19	−2	−2	0	−2
31	The guidance was clearly communicated at all stages	−4	−4	1	−3
32	In responding to COVID‐19, the government should have listened to experts sooner	5	−1	−1	0
33	No‐ones safe until everyone is vaccinated	1	−6	2	−1
34	A lack of trust in the government means people don't follow restrictions or listen to the guidance	2	3	0	1
35	The government has had a clear strategy on how to deal with the pandemic	−4	−5	3	−4
36	The government decided to sacrifice some jobs for the sake of others	−1	2	−1	0
37	Government financial support protected their citizens from the effects of the pandemic	−1	−2	3	1
38	Local areas should have had more control over what restrictions were implemented	−1	1	−2	−1
39	The pandemic is just a way for the government to reduce our freedoms	−6	0	−6	−6
40	The government should be held to account for their poor decisions	5	3	−3	1
41	*People who struggle with technology found it difficult to cope during the pandemic*	2	1	2	3
42	The pandemic is the least of people's problems	−4	−1	−4	−2
43	It is wrong for rich countries to stockpile COVID‐19 vaccinations	3	0	1	5
44	The virus should have been contained at its source in China	−2	2	−4	−3
45	The World Health Organisation should have done more to stop the spread of the virus in the early days	−1	1	−2	−2
46	Border control for COVID‐19 has been a success	−5	−5	−2	−5
47	*The guidance has changed so frequently I don't know what is safe anymore* a	−1	−1	−2	0
48	I trust the vaccines	4	−5	4	4
49	The pandemic has become politicised	1	4	3	1
50	The response to COVID‐19 shows that the world can take drastic action when faced with a global threat	0	−1	6	4
51	Vulnerable people have been supported more by family, community action, and/or charities than by the government	2	2	−1	2
52	It was unfair to sacrifice children's development by closing schools for older people's health	−2	4	−4	−2
53	The whole pandemic is a conspiracy	−6	−3	−6	−6
54	COVID‐19 has exposed the deep‐rooted racism that exists in society	1	−2	−3	3
55	Staying inside is the wrong response to a new virus—the government should encourage safe ways to be outside	−3	2	0	−2
56	I'd rather take my chances than risk the side effects of the vaccine	−5	4	−5	−5
57	People should have to show they are fully vaccinated in order to travel or attend large‐scale indoor events	1	−6	2	−1
58	There are other ways to tackle COVID‐19 other than relying on vaccines	−2	3	−2	−3
59	It's ok to break lockdown as long as you don't put anyone else at risk	−4	−1	−5	−3
60	Existing inequalities meant people from ethnic minorities suffered more than others in society	3	0	0	5

*Note*: *Italics* indicate consensus statements.

a Distinguishing statements.

**Table 2:** Factor loadings and participant characteristics.

ID	Occupation	Experience/background	Gender/age/government	Data collection	Factor 1	Factor 2	Factor 3	Factor 4
*Factor 1*								
FC003	Nurse—mental health services	Worked in care at the beginning of the pandemic	F/18–30/United Kingdom	In person	**0.8032x**	−0.1302	0.1963	0.3279
FC011	Junior doctor	Hospital‐based doctor—working throughout	F/31–50/United Kingdom	Online	**0.775x**	0.1486	0.1899	0.2052
FC001	Unemployed	Shielding and low income, friends have died from COVID‐19	M/51–64/Scotland	In person	**0.7486x**	−0.0196	0.1365	0.1877
FC044	Operations manager	Working in events, furloughed, had COVID‐19	F/18–30/‐	Online	**0.7471x**	0.1998	0.2995	0.1042
FC034	Researcher on COVID‐19	Working on government research, respiratory condition	F/31–50/United Kingdom	Online	**0.7331x**	0.1262	0.1607	0.0963
FC025	Retired	Volunteered with mutual aid group during lockdowns, shielding	F/65+/United Kingdom	Online	**0.7158x**	−0.0721	0.2919	0.2212
FC059	Professor of public policy	Member of an advisory group on COVID‐19	M/65+/United Kingdom	Online	**0.6886x**	−0.0892	0.0372	0.3126
FC031	Support worker—personal assistant	Low income, furloughed during COVID‐19, family member shielding	F/31–50/Scotland	Online	**0.6674x**	−0.11	0.3443	0.1332
FC026	Associate academic registrar	Helped family/friends during COVID‐19	F/51–64/United Kingdom	Online	**0.6548x**	0.3191	−0.1109	0.3216
FC040	PhD student	BAME, unable to travel to see family during the pandemic	M/18–30/United Kingdom	In person	**0.6416x**	0.1821	−0.007	−0.11
FC005	Health worker	Worked with intensive care patients during lockdowns	F/18–30/United Kingdom	In person	**0.639x**	0.3561	0.0439	0.2391
FC016	Shop assistant	Working in the Food Sector/Essential worker	F/65+/United Kingdom	In person	**0.6287x**	0.0342	0.2566	0.158
FC004	Health worker	Lost job due to COVID, travelled internationally between lockdowns, worked in healthcare during COVID‐19	F/18–30/United Kingdom	In person	**0.6158x**	0.19	0.1826	0.3178
FC056	Voluntary support worker	BAME, low income, shielding, family member died from COVID‐19	F/51–64/United Kingdom	In person	**0.5869x**	−0.0325	0.3472	−0.0385
FC013	Professor of social policy	Working in academia and contributed to citizen panel research on COVID‐19	M/31–50/United Kingdom	Online	**0.5808x**	−0.0237	0.4405	0.2015
FC033	Retired	Shielding, retired	F/65+/Scotland	In person	**0.5775x**	0.0599	0.1091	0.3874
FC043	HR consultant	Shielding, made redundant during COVID‐19	F/31–50/United Kingdom	Online	**0.5692x**	0.0191	0.3646	0.2761
FC019	Artist	Low income, carer, volunteered during the pandemic	F/51–64/United Kingdom	Online	**0.5624x**	0.1344	−0.0661	0.0837
FC002	Equality diversity and inclusion official	BAME, family shielding, worked from home	F/18–30/United Kingdom	Online	**0.5217x**	−0.1112	0.2569	0.2986
FC062	Charity case manager	Shielding, family member died from COVID‐19	M/31–50/United Kingdom	Online	**0.3893x**	0.1043	0.2204	0.0735
FC053	Social care support worker	Undergraduate during COVID‐19	F/31–50/United Kingdom	In person	0.5168	0.1503	0.4192	0.3045
FC050	Journalist	Sub‐editor, furloughed	F/18–30/United Kingdom	Online	0.502	0.4698	0.0958	0.2056
FC020	Research officer	Social policy, volunteered during the pandemic	F/31–50/Scotland	In person	0.4811	−0.0186	0.3735	0.436
FC058	Associate director	Health focussed organisation, friends have died from COVID‐19	M/31–50/United Kingdom	Online	0.4796	0.3217	0.2674	0.2473
FC066	Medical director	Working in public health	F/‐/United Kingdom	Online	0.4084	0.1427	0.0834	0.394
*Factor 2*								
FC024	Retired	Against the lockdowns, one dose of vaccine, family member shielding	M/65+/United Kingdom	Online	−0.0103	**0.8272x**	−0.1757	0.0036
FC021	Nurse	Working in public health	F/31–50/‐	Postal	−0.0577	**0.7132x**	−0.0956	0.007
FC067	Unemployed	Unemployed	M/31–50/United Kingdom	Online	0.0961	**0.7091x**	0.1559	0.0452
FC048	Beauty therapist	Gave birth during COVID‐19, self‐employed, made redundant during COVID‐19	F/18–30/United Kingdom	In person	0.0194	**0.6756x**	−0.25	0.218
FC028	Swim coach	Furloughed during lockdowns, against lockdowns	M/18–30/United Kingdom	In person	−0.0134	**0.6639x**	−0.2094	−0.109
FC022	Driver and stage manager	Against the vaccines, changed job during COVID‐19	M/31–50/‐	Postal	−0.4299	**0.6567x**	−0.2987	−0.1869
FC060	Professor of epidemiology	Infectious disease expert	F/51–64/United Kingdom	Online	0.1478	**0.6449x**	−0.0252	0.3923
FC061	Personal trainer	Family member died from COVID, full‐time student (undergraduate) during COVID‐19	M/18–30/United Kingdom	Online	0.3682	**0.5666x**	0.1653	0.1335
FC054	Warehouse operator	Undergraduate during COVID‐19, made redundant	F/18–30/Scotland	Online	0.2097	**0.5247x**	0.3157	0.1879
FC036	Unemployed	BAME, friends have died from COVID‐19	F/51–64/United Kingdom	In person	0.062	**0.404x**	−0.0416	0.0782
FC035	Retired	Family member died from COVID‐19, BAME	F/51–64/United Kingdom	In person	0.3192	**0.3711x**	−0.16	0.1009
*Factor 3*								
FC047	Occupational Therapist	Family member shielding during COVID	F/31–50/Scotland	In person	0.3719	−0.0635	**0.7733x**	0.0704
FC014	Senior public health official	Working in public health	M/51–64/Scotland	Online	0.0933	−0.2987	**0.711x**	0.1752
FC052	Youth worker	Made redundant, shielding	F/65+/United Kingdom	Online	−0.0819	−0.1082	**0.6545x**	0.1042
FC064	Flour grinder	Family member died from COVID, works in food production	M/31‐50/United Kingdom	Online	0.261	0.0794	**0.6351x**	0.0769
FC015	Process operator, production	Production (Pharmaceutics)	M/31–50/United Kingdom	In person	0.3586	−0.0713	**0.6241x**	0.1387
FC007	Teacher (secondary school)	Family member shielding during COVID	F/31–50/United Kingdom	In person	0.2571	−0.0405	**0.5928x**	0.3986
FC063	Dog walker	Changed job during COVID	M/51–64/United Kingdom	Online	0.058	−0.2424	**0.5195x**	0.2527
FC046	Senior public health official	Working in public health	F/‐/United Kingdom	Online	0.3945	−0.169	0.5122	0.2913
*Factor 4*								
FC010	At‐home carer	Caring for son who was shielding. Shielding herself	F/65+/United Kingdom	In person	0.5099	0.0671	0.0894	**0.6948x**
FC012	Clinical lecturer	Worked for public health authority during the pandemic	F/31–50/United Kingdom	Online	0.4873	0.1844	0.342	**0.6503x**
FC006	Headteacher (secondary school)	Volunteered during COVID (Foodbank)	F/31–50/Scotland	In person	0.3186	−0.0451	0.402	**0.6482x**
FC023	Infectious diseases researcher	Working in academia and public health	F/65+/United Kingdom	Online	0.3531	0.1359	0.4081	**0.587x**
FC008	School teacher (depute head)	Working in education/essential worker	F/31–50/United Kingdom	In person	0.2354	0.2199	0.3925	**0.5438x**
FC055	Associate director	Career in mental health, family member shielding	F/31–50/Scotland	Online	0.4345	0.0784	0.4254	0.4899
FC032	Professor of public policy	Family member passed away from COVID	M/51–64/United Kingdom	In person	−0.0243	0.292	0.1615	**0.4845x**
FC057	Journalist	Focusing on health, science and social policy	F/31–50/United Kingdom	Online	0.3475	0.4733	−0.064	0.4816
FC030	Clinical lecturer	Working in academia and public health	M/31–50/United Kingdom	Online	0.1534	0.361	0.2137	**0.4703x**
FC027	Charity communications officer	International student during the pandemic	M/31–50/United Kingdom	In person	0.2503	0.0706	0.3167	0.3547
			Explained variance		22%	11%	11%	10%

*Note*: ‘FC’ is part of the participant identifier for this study. A single dash shows no response. The factor loadings of defining card‐sorts are indicated with an X and in bold text. Defining card‐sorts meet two criteria: (i) the loadings are statistically significant (*p *< .05). The significance level is calculated as .96 × (SE). SE represents the standard error that is defined as 1/√*N*, where N is the number of statements in the statement set. 1.96 × (0.13) = 1.96 (1/√60) = 0.25. (ii) The factor loading ‘explains’ more than half of the common variance. Common variance refers to how much an individual sort holds in common with the other factors and whether or not this is significantly different.

Abbreviations: M, male; F, female.

### 2.1 The statement set

In a Q methodology study, the starting point is to locate a wide range of ‘conversational possibilities’^40^ on the topic in question to develop the statements that are ranked in the card‐sort. To access conversations on perspectives of COVID‐19 and COVID‐19 policies, we utilised different sources, collecting data in a variety of ways to ensure that we were covering as much of the conversation as possible. Grey literature provided a rich source of potential statements as these were often informed by participants who were experiencing the pandemic first hand and telling their stories qualitatively. For example, we extracted statements from the Scottish Government's Citizens' Panel on COVID‐19,^41^ daily diaries on living during the pandemic^42^ and the Carnegie UK report from their community listening project^43^ as they represented recent and rich sources where different perspectives on the pandemic were expressed. We extracted over 200 statements across these documents.

Extracted statements were then coded descriptively, which yielded 47 different themes (such as freedom, religion and recovery from disease); these were also coded by three different levels of impact: individuals, communities and society. From the 200 statements, duplicate and similar statements were then removed and similar statements were merged while, as closely as possible, retaining the naturalistic wording of original statements. Two rounds of piloting, with eight people in total, led to some changes in wording, for example, from ‘I don't trust the vaccines’ to ‘I trust the vaccines’ (statement 48), to avoid the possibility of double negatives. At the end of each pilot interview, participants were asked if anything was missing that they would like to see added to the set. Our final statement set consists of 60 statements (see Table 1) that covered our identified themes and research question. There is no typical number of statements in a Q study. However, most contemporary Q studies tend to fall between 40 and 60 statements.^39^ The number of statements in a study is driven by the number and range of views expressed on the topic. As there were a wide range of views on the COVID‐19 pandemic (perhaps because it touched so many aspects of everyday life), we had a relatively large number of statements. Table 1 below provides the set of 60 statements and, in the rightmost columns, their position on an idealised grid (see Appendix S1) for each factor.

### 2.2 Timing and policy context

**Figure 1:**
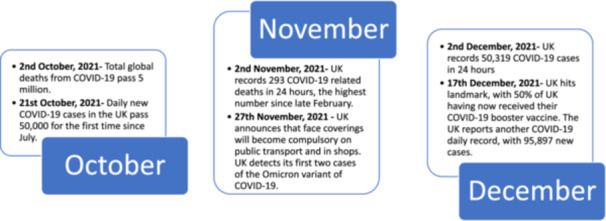
Timeline (months of data collection).

Data were collected from early October 2021 until late December 2021. As the pandemic changed quickly, Figure 1 shows key moments during this time period and deaths attributed to Covid‐19.

In the United Kingdom, restrictions on movement, together with the closure of public services and businesses in March 2020, and again in November 2020, had an impact on citizens' freedoms. This initial and sudden disruption to lives was followed by public health advice that aimed to protect the health system and the lives of at‐risk groups, such as older people or those with existing health problems.

### 2.3 Participant sample

We recognise that public(s) can mean many things, and that many different public(s) assemble, or are assembled, for different reasons. We are all members of the public as well as sometimes patients, professionals, politicians and so forth.^44^, ^45^, ^46^ In this study, and following conventions in Q methodology, participants were sampled because they were likely to express rich and distinct views on the COVID‐19 pandemic. It is not, therefore, intended to be a ‘general public’ sample as might be typically understood, and it is not representative of ‘the general public’, but rather a purposefully selected sample of people with a wide range of different experiences and expertise that are likely to be pertinent to their views on COVID‐19.

We recruited people living in Scotland and England because the UK and Scottish Governments, arguably, took somewhat different approaches to restrictions and to communication at different times during the pandemic. Scotland and England also have different health policy environments, as health is a devolved matter in Scotland. We recruited health practitioners, health and social policy academics, researchers and relevant policy actors, key workers, furloughed staff (those working in jobs that could not be worked from home, with salaries subsidised by the government so that they could remain at home while retaining their jobs), individuals directed to ‘shield’ (isolate due to particular health conditions or vulnerabilities) by the UK National Health Service, people with different vaccination statuses and self‐reported political affiliations.

Initially, we recruited participants through two approaches. First, we leveraged our personal and professional networks to identify individuals who met our criteria: for example, those we knew had been furloughed due to COVID‐19 or who actively expressed viewpoints on social media regarding the pandemic. Second, we contacted public figures known for holding specific viewpoints. After these initial approaches, we used snowball sampling to reach other participants; following each completed card sort, participants were asked to provide contact details of other individuals who they believed would have rich and different views. In the later stages of the study, we used a recruitment agency to identify individuals with different characteristics and profiles who were not yet well covered in our sample; these included people who were based in England, male, had low education/income, those with a conservative political affiliation and unvaccinated. While the most important aspect of our sample was the variety of viewpoints that we were collecting, we also wanted to ensure that we covered a broad range of demographic profiles that could be associated with different views. For example, people who chose not to receive a vaccine may have had a different point of view than people who were vaccinated.

Potential participants were then invited by email to take part in the study, with a project information sheet attached (see Appendix S3). Upon agreement to take part, the participants were sent a consent form electronically (see Appendix S4), which they would then respond to via email before data collection took place. This process included the two postal sorts. For the face‐to‐face card‐sorts, paper copies of these documents were provided and completed in person.

The size of samples in Q studies varies, but is usually similar to qualitative interview studies, or perhaps a little larger, with 40–60 participants usually being sufficient, at which point additional participants often serve only to shore up a shared view point, but do not add a new perspective (this is akin to notions of data saturation in qualitative methods). Our 54 participants were asked, after their card‐sort (or during if they brought it up as an issue), whether they were thinking about the UK Government approach or the Scottish Government approach to handling the pandemic when they were sorting their cards as this may have impacted upon their understanding and placements of statements (clearly, this was only an option for people who came from Scotland, as they were exposed to both governments; those in England would only be subject to the UK Government. See Table 2 for individual responses). Forty‐two participants sorted their cards according to the UK Government, 9 according to the Scottish Government and 3 did not provide an answer.

### 2.4 Data collection

Data were collected using three elicitation methods; online, face to face and postal. Online data were collected using QMethodSoftware,^47^ which is a dedicated online Q methodology data collection platform. We uploaded the statements and grid in the software before multiple rounds of internal testing amongst the research team, followed by two rounds with our pilot participants. All online card‐sorts were conducted with the help of a researcher, using Microsoft Teams. Participants were asked to share their screens so that the researcher could guide the card‐sort process, answer questions and monitor for any difficulties. In parallel, the same statement set was designed, tested and printed for use in person by the research team. During the face‐to‐face card‐sorts, social distancing was observed and face masks were worn to limit risk of infection for the researcher and the participant. The postal card‐sorts and written instructions were designed by the research team, tested and then sent to participants via recorded mail. Respondents also received a prepaid envelope so that they could return their response free of charge, and were provided with mobile numbers and email addresses to contact the researchers if they needed support. Both of the postal responses provided written comments on their sort. While there were limitations to these approaches, we took the view that obtaining data in different ways was better than excluding groups who could not be reached face to face (due to geography, health or work commitments). A mixed approach to data collection has been tested in previous studies and has not been found to impact responses.^48^

Respondents across all three modes of data collection were presented with a shuffled set of 60 statement cards (see Table 1) and then asked to consider each statement in turn according to the sorting instruction: ‘My view on the COVID‐19 pandemic’. Respondents initially sorted the statements into one of three piles: ‘most unlike my point of view’, ‘neither like nor unlike my point of view’ and ‘most like my point of view’. Respondents were then instructed to further sort the statements onto a bell‐shaped grid working from the outer edges towards the centre of the grid (see Appendix S1 for grid).

Following the placement of all the statements, a postsort interview was conducted with the participant where they were asked specific questions about statements at the extreme ends of their grid, statements of interest throughout the grid and open‐ended questions about how they made choices between statements. This short interview was audio‐recorded and transcribed verbatim. These qualitative data were used to aid the selection and interpretation of the factors.

## 3 ANALYSIS

Data from the card‐sorts were analysed using KADE software.^49^ Following Watts and Stenner,^39^ a centroid factor analysis was followed by Varimax rotation to identify a small number of shared viewpoints (factors) based on the correlations between respondents' card‐sorts. While the statistical analysis is valuable for exploring clusters of similarity in a quantitative manner, in Q factor analysis, generally, a greater emphasis is placed on the qualitative accounts in selecting a solution and interpreting the factors.

Quantitatively, selecting a factor solution (identifying a number of shared viewpoints) in Q methodology requires that at least two card‐sorts define a factor. In this study, a defining card‐sort was one with statistically significant (*p* < .05) factor loading (the level of correlation between the card‐sort and the factor) and majority common variance (see the bottom of Table 2 for a fuller explanation of these criteria). These basic requirements will usually produce more than one possible solution, and all possible solutions are then examined in terms of both statistical qualities and qualitative data. Characterising statements are those that are placed near the extreme ends of the grid for each idealised card‐sort (−6, −5, +5 and +6 on a grid ranging from −6 to +6). Distinguishing statements are those positioned significantly differently in one factor than the same statement in the other factors. Consensus statements are those that are not positioned significantly differently between any pair of factors.

Qualitatively, arriving at a factor solution requires that factors must be interpretable in relation to the narratives provided by the participants during their postsort interview. Factors are considered in terms of whether they make sense theoretically and in terms of their internal consistency. The data provided in the interviews (particularly from those with defining card‐sorts) help to enrich the factor interpretation, and in the factor descriptions, respondents' quotes are used to explain the point of view.

### 3.1 Results

Twenty‐two card‐sorts were completed face to face, in person with one of the authors; 30 card‐sorts were completed online; and two card‐sorts were completed via post. Table 2 shows the four‐factor solution that satisfied qualitative and quantitative criteria. All card‐sorts were significantly correlated with one of the factors (there were no ‘null loaders’); 45 card‐sorts were flagged as defining one of the four‐factors. Twenty card‐sorts define Factor 1, 11 sorts define Factor 2, 7 sorts define Factor 3 and 7 sorts define Factor 4.

### 3.2 Factor descriptions

The factor descriptions below are abbreviated from longer, annotated accounts, attached in Appendix S2, which refer to specific statement numbers and include participant quotes.

#### 3.2.1 Factor 1—Dangerous and unaccountable leadership

Factor 1 describes a view that the government should have been held to account for their inept and dangerous response to handling the pandemic that made a bad situation worse. The seriousness of COVID‐19 required a commensurate response from the government. However, the government's lack of a clear strategy and slow response on how to deal with the pandemic cost lives and impacted people's mental health. The government failed to listen to experts or learn from experiences in other countries. This meant that lockdowns did not happen quick enough to prevent devastating impacts. Then, when the government made a decision, it was undermined by poor implementation and communication, for example, around its track and trace system and border control, or actions that eroded trust and impacted on people's compliance with rules.

This inept government response was compounded by a lack of funding for social care services, leading to an overreliance on the NHS, which lacked resources and was not fit for purpose. This meant that people were dying from preventable illnesses because our systems were not strong enough to cope with the pandemic. Despite a lack of trust in the government, there was great trust in the vaccines, which brought relief and a way out of the pandemic. However, the United Kingdom should not have been stockpiling vaccines. Besides this being morally wrong, it increased the risk and worry of new variants emerging from countries with low vaccination rates.

It was imperative that the government were held to account for their poor, and at times dangerous, decisions. They should have been doing everything in their power to keep people safe but this was clearly not the case. Their failure to act meant that those who were already in vulnerable positions were hit hardest.

#### 3.2.2 Factor 2—Fear and anger at policy and public responses

Participants defining Factor 2 were deeply concerned that the portrayal of, and the response to, the virus both by the government and by members of society led to preventable deaths, unnecessary harms and devastating impacts on people's mental health.

The government and media created fear in society, and a culture in which uncritical compliance was seen as a virtue with no room for alternative views. Friends, family, neighbours and acquaintances became authoritarian during the pandemic, making ridiculous requests, such as washing the shopping or using hand sanitiser, and neighbours started to act like secret police.

Decisions such as lockdowns were unfair and dangerous, causing unnecessary harm, perhaps even more than the virus, particularly to poor inner‐city children. Generally, children were not vulnerable to the virus, which made closing schools particularly unfair; it had a devastating impact on young people's education and mental health, particularly as some had no other way to escape difficult home lives. Staying inside was an easy way for the government to cover for an underfunded NHS, rather than a way to keep people healthy. Yet, despite such restrictions, people continued to catch COVID‐19 from going into hospital with other illnesses and died from preventable illnesses.

There was a lot of misinformation around COVID‐19; contradictory government guidance created uncertainty about what to do, impacting mental health and leading people to listen to alternative sources of news. Track and trace systems did not work, only enriching those with connections to the establishment through lucrative government contracts. The government failed to protect all their citizens from the financial effects of the pandemic and did not pay essential workers what they deserved. While international organisations, such as the World Health Organisation, could have done more to stop COVID from spreading, the government failed its citizens with poor border control. At the same time, it was ridiculous that you would need proof of vaccination to attend large‐scale indoor events.

The approach to vaccinations was emblematic of the overall response to the pandemic as what should have been a solution was more of a problem. Instead of bringing relief to people, there was suspicion, worry and mistrust because of the speed and processes of vaccine development and roll‐out, pharmaceutical companies being profit driven and concerns of short‐ and long‐term side‐effects. While vaccines may have been a risk worth taking for those who are vulnerable from COVID‐19, they did not prevent people from spreading it and vaccinations also came with health risks.

#### 3.2.3 Factor 3—Governing through a crisis

For Factor 3, the COVID‐19 pandemic was an unprecedented global tragedy causing difficulties for governing but there were some positive consequences.

The COVID‐19 pandemic required drastic action, global cooperation and unprecedented policy choice. As in other major crises, such as the World Wars, governments made mistakes that costed lives. However, judging these decisions is only possible on retrospection. Those who used the government's performance against them do so for political gain and the media sometimes created witch hunts against certain officials.

Governments had clear political and clinical strategies on how to deal with the pandemic and adapted to new learning and information. Tough decisions needed to be made. Responses to the pandemic, such as lockdowns, had a devastating effect on people's mental health and led to loneliness, family separation and harms to education. However, these decisions were not unfair; the priority was to reduce deaths and infection. Other countries, such as China, or organisations like the World Health Organisation were not responsible for the spread of the virus. Learning from the pandemic would also help with planning for the next time it happens.

The pandemic, while devastating, highlighted areas of positivity across different spheres of society. The rapid development and distribution of safe vaccines showed how public and private partnerships can form on a national and global scale to tackle a global threat. While social media spread disinformation and misinformation, in reality, vaccines saved more lives globally than any other single healthcare intervention in history and gave people their lives back. Making sure that everyone around the world was vaccinated helped keep us all safe from variants. From an individual perspective, the pandemic made us reassess how we live and work and interact with each other for the better.

#### 3.2.4 Factor 4—Injustices exposed

COVID‐19 exposed inequalities at the individual, societal and global levels like nothing before. At the individual level, the low pay of essential workers was a gross injustice that needed to change as we were completely reliant on them during the lockdown. Vulnerable essential workers were more supported by family, the community or charities than the government. We should financially compensate essential workers to reflect how much we value them as a society. While pretty much everyone would have caught COVID‐19 in the end, the effects of the pandemic were felt disproportionately by those who were poorer. Being richer insulated you and meant that you were more likely to use technology to keep your social life and job going online.

At the societal level, existing inequalities meant that people from ethnic minorities suffered more than others in society, with deep‐rooted racism exposed. The government let down these groups and others with existing vulnerabilities, who instead had to rely on support from family, community action or charities. This community ethos was positive but the government should have done more.

At the global level, richer nations were able to better protect themselves from the effects of the virus and stockpiled COVID‐19 vaccinations. Vaccines were the best way to tackle COVID‐19 and their development brought a sense of relief but stockpiling exposed a lack of care for the whole of society and humanity.

The pandemic was unparalleled, with devastating impacts on health and mental health. Governments were trying to respond to an unprecedented act of nature but the overall strategy and communication were poor and restrictions, such as border control and track and trace, were not a success. In contrast, the general public, by and large, behaved well and supported each other. The government should not force people into following guidelines; instead, people should, in most areas, have been allowed to use their common sense and get on with things.

## 4 DISCUSSION

The four perspectives found in this study on the COVID‐19 pandemic demonstrate the plurality and nuance in perspectives that exist on this topic, going beyond a binary narrative that has been common in popular and social media. The first perspective highlights a lack of trust in the UK Government and describes the poor decisions made in managing the COVID‐19 response. Those holding this view followed the rules and expressed anger at those who did not. Factor 2 sees the measures put in place to tackle the pandemic as raising fear in order to control the population. Government, media and societal responses led to preventable non‐COVID deaths and other societal harms. The third factor describes a perspective more supportive of government response. COVID‐19 was a new and emerging disease and limited information was available to governments on which to make decisions. The perspective expressed by Factor 4 is concerned with how COVID‐19 highlighted and widened inequalities within society. Identifying and describing these perspectives help move beyond a polarised discourse surrounding COVID‐19 and provide a richer narrative from different perspectives. We discuss below areas of common ground and divergence between the four perspectives, with a view to moving from polarity to plurality and to enhancing future debates.

### 4.1 Common ground and divergent views

Importantly, there is agreement across all perspectives that the pandemic was not a conspiracy as statement 53 ranked consistently in the negative columns across all factors (see Table 1). Similarly, all accounts are concerned with the effects on people's mental health (statement 18) and that it was a tragedy for people to be dying of preventable illnesses (statement 2). As a starting point, this indicates that there is shared recognition of the problem and the seriousness of it.

Different perspectives that appear to have things in common on the surface do not always share the same motivations. This is seen most clearly regarding views on the UK Government's handling of the pandemic. While the account described in Factor 3 is more sympathetic, the other views are critical of government strategy and the accounts described in Factors 1 and 2 both express distrust of governments, who they believe made poor decisions, and yet, the rationales in Factors 1 and 2 are quite different. Those associated with Factor 1 acted to keep themselves and others safe by following guidance and were critical of contradictory advice, failed policies and those who flouted rules. Those associated with Factor 2 were critical of the government's restriction of freedoms and control over people, particularly in relation to evidence for vaccination, lockdowns and school closures. They are appalled by the levels of unquestioning obedience. This erosion of trust in decision‐makers is likely to impact on compliance.

Arguably, a functioning democracy requires the public to trust its elected officials.^3^ However, the consequences of mistrust are particularly stark when dealing with an infectious public health emergency. Importantly, data collection finished before the ‘Partygate’ scandal in the Westminster Government that ultimately led to the downfall of the UK Prime Minister, which is likely to have led to further distrust in the government.^50^

While very different views are expressed in the descriptions of Factors 2 and 3, there was agreement on the negative impact of the media on, and the politicisation of, the pandemic. Engaging in a form of critical debate is needed to make better decisions, but when this is reduced to political point scoring or generates hysteria, paranoia and worry in the context of commercial media outlets, who make money through media consumption, there are potentially negative consequences.^51^ Again, this is seen in the tone of Factor 2 and the confusion and uncertainty caused by the amount of misinformation and contradictions around COVID‐19 and the responses to it.

The vilification of those expressing opinions contrary to the mainstream meant that instead of engagement and debate, they were ‘othered’ and labelled ‘anti‐vaxxers’. While the labelling of our factors could be misconstrued as another mechanism that supports the ‘Us vs Them’ mentality, we reveal nuance that we believe enhances understanding and, in some ways, are seeking to soften the hard lines between the accounts. It is important to note that no individual is a perfect representation of Factor 1, for example, because they are likely to have a factor loading (correlation coefficient) less than 1 and also to associate (in a weaker manner) with one or more other factors (see Table 2). This reinforces our observation that while these distinct narratives exist, and people tend to align with one factor more than another, there are areas of common ground. Understanding of these might be used to better manage communication in future public health crises.

### 4.2 Contribution to wider literature

This study complements the existing quantitative and qualitative studies on public preferences and perspectives towards COVID‐19 in a number of ways. For example, a preference‐based study by Sabat et al. conducted in April 2020^52^ found large regional and age‐related heterogeneities in levels of trust and policy support. Amongst other measures, the authors call for a ‘tailoring of messages and means of communication to specific groups of the society’.^52^ ^,p.917^ Similarly, Chorus et al.^24^ find high levels of heterogeneity within their respondent sample with regard to the weights attributed to different policy impacts. Our study provides information about different shared perspectives that might go some way towards understanding heterogeneity in preferences in future studies. So far, only a limited number of qualitative papers exist on COVID‐19. Amongst these studies, none covers as broad a sample as our study, which featured, for example, experts helping to lead the pandemic response, key workers being exposed to the virus itself and those who were disadvantaged during the pandemic due to their circumstances. Gaining a wide range of views is important because the pandemic impacted upon everyone in different ways, and ensuring that we capture those perspectives may help in addressing future policy concerns during a pandemic in the United Kingdom.

### 4.3 Strengths and limitations

This study was conducted in late 2021, by which point people had experienced at least one lockdown and the introduction of vaccines; this longer time frame adds new information to our understanding of public responses to the pandemic compared to the large number of early public preference studies. Though perspectives may have shifted since conducting this research, our findings offer a valuable snapshot of public views during the height of the pandemic. This period was marked by experiences with the COVID‐19 virus itself, evolving restrictions on daily life and a growing awareness of potential harms. However, despite this time frame, because responses to the pandemic were changing frequently, it is possible that we did not capture all issues that could have been relevant to the population when designing our statement set.

Recognising the difficulties of conducting primary research during a pandemic, data collection was in person, online and by post based on the participant's preference. We took the view that providing means to gather data in different ways was preferable to excluding groups who could not meet face to face. We acknowledge that there may have been some differences across methods of data collection that could conceivably affect responses, such as social acceptability bias in those card‐sorts with the researcher present. However, our results do not indicate that there was a link between the form of data collection and the association between participants' card‐sorts and the identified factors (see Table 2). For participants completing online without a reliable internet connection or by post, it was not possible to complete a follow‐up interview to aid our interpretation of the factors, and for these participants, we relied on written comments only. Our study describes perspectives in rich detail but does not go so far as to connect those with policy choices or preferences over policy attributes, though there is scope for such work in the future. Equally, research comparing perspectives across other countries, with different experiences and government responses, would be very interesting. Lastly, our study says nothing about how common these views are across the population. Q‐to‐Survey methods could be used to explore the prevalence and distribution of views.^53^

### 4.4 Conclusion

The findings from this Q methodology study provide new evidence that can be used not only in understanding the response to COVID‐19 but also in preparing for future public health campaigns. While there are always going to be different views, understanding where there are areas of agreement, as well as discord, can help to build social cohesion and foster a sense of resilience amongst individuals, communities and societies in the face of future crises. We view this work as a starting point regarding what views exist and where areas of similarity and difference lie. Societal and community resilience, a key part of the UK COVID‐19 enquiry, depends on high levels of social cohesion that can be nurtured by understanding what we have in common with those different to ourselves and enhancing the quality of discussion and debate. By exploring these views, we can move towards a more nuanced approach to plurality and polarity.

## AUTHOR CONTRIBUTIONS

**Jack Rendall**: Investigation; methodology; writing—original draft; writing—review and editing; visualisation; validation; software; formal analysis; project administration; data curation. **Neil McHugh**: Methodology; conceptualisation; investigation; writing—original draft; writing— review and editing; validation; supervision; project administration; formal analysis; data curation. **Rachel Baker**: Methodology; writing—original draft; writing—review and editing; formal analysis; supervision; validation; conceptualisation. **Helen Mason**: Methodology; conceptualisation; writing—original draft; writing—review and editing; validation; formal analysis; supervision. **Olga Biosca**: Funding acquisition; conceptualisation; writing—review and editing; writing—original draft; project administration; supervision; data curation.

## CONFLICT OF INTEREST STATEMENT

The authors declare no conflict of interest.

## ETHICS STATEMENT

Ethical approval was granted from the Glasgow School for Business and Society Research Ethics Committee and Glasgow Caledonian University Ethics Committee [GSBS EC017]. All participants in the manuscript provided written informed consent ahead of their participation in the data collection, both for the card‐sorting data and the postsort interview data.

## Supporting information

Supporting information.

Supporting information.

Supporting information.

Supporting information.

## Data Availability

The data that support the findings of this study are openly available in the UK Data Repository catalogue at https://reshare.ukdataservice.ac.uk/855895/.
