# Supplementary figures and images for: From polarity to plurality: Perceptions of COVID‐19 and policy measures in England and Scotland
Source: Health Expect. 2024 May 11;27(3):e14069. doi: 10.1111/hex.14069 (PMC11087883; doi:10.1111/hex.14069)

Appendix 1 – Q Sort Grid


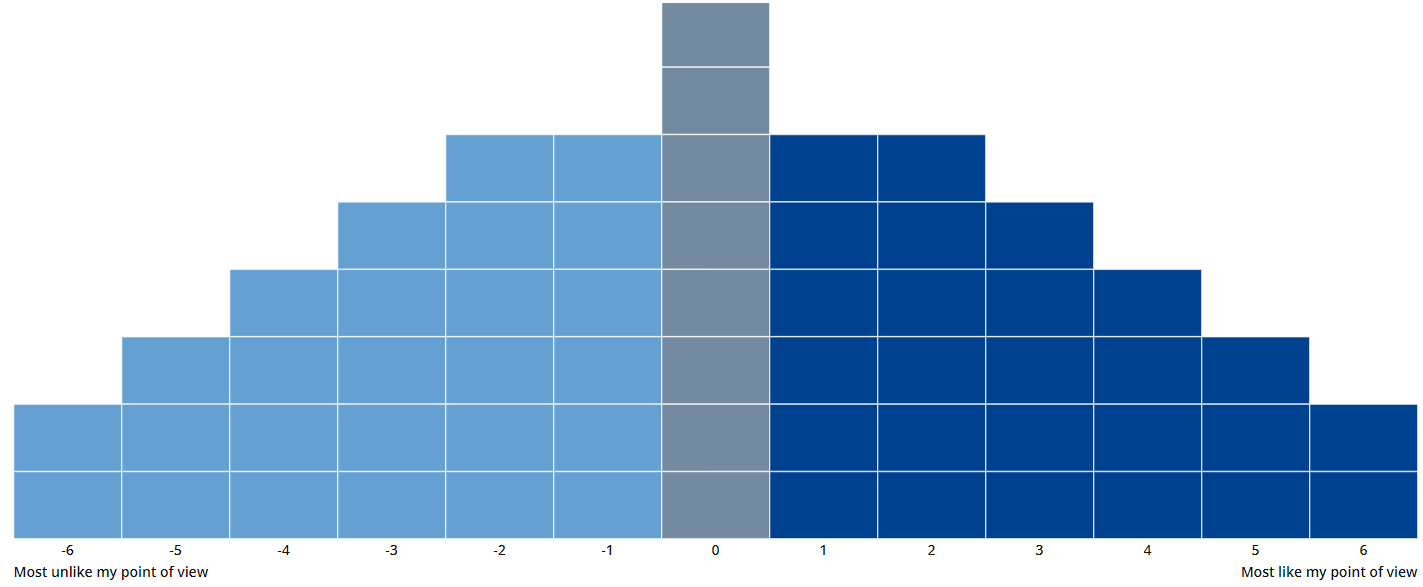

Supplement: Supplementary file 1 — Supporting information. [file HEX-27-e14069-s003.docx]
