# Supplementary material for: From polarity to plurality: Perceptions of COVID‐19 and policy measures in England and Scotland
Source: Health Expect. 2024 May 11;27(3):e14069. doi: 10.1111/hex.14069 (PMC11087883; doi:10.1111/hex.14069)
Supplement: Supplementary file 2 — Supporting information. [file HEX-27-e14069-s004.docx]

## Appendix 2 – full factor descriptions with statement references and quotations from defining participants

These longer form factor descriptions (which are abbreviated in the paper) are described using the following notation: # indicates the statement number and +/- the position of the statement on the grid (see Appendix 1). For example (#10, +6) means that statement number 10 was placed in position +6 for the factor in question. Quotes from defining participants for each factor are shown in italics followed by participants’ unique ID numbers (e.g. FC057) as listed in Table 2.

## Factor 1 – Dangerous and unaccountable leadership

Factor 1 describes a view that wanted the Government to be held to account for their inept and dangerous response to handling the pandemic that made a bad situation worse (#10, +6; #18, +6; #25, +5; #32, +5; #40, +5; #12, +4; #46, -5; #35, -4; #31, -4; #26, -3; #14, -3).

The seriousness of COVID-19 required a commensurate response from the government (#53, -6; #39, -6; #1, -5; #42, -4). However, the government’s lack of clear strategy and slow response on how to deal with the pandemic cost lives and impacted people’s mental health (#18, +6; #5, +5; #4, +3; #35, -4). The Government failed to listen to experts or learn from “*how the pandemic was developing in the Far East, in China*” (FC025) or “*other countries that were ahead of us”* (FC005). This meant lockdown did not happen quick enough to prevent devastating impacts (#25, +5; #32, +5). Then when the Government made a decision, it was undermined by poor implementation, poor communication or actions that eroded trust (#31, -4; #34, +2). For example, the government’s system to track and trace those with infections and border control did not work well (#46, -5; #14, -3) and “*…the hypocrisy of what they will tell the general population to do and then what they will do. Scooting around the country when they tell other people not to do that. So, they will say one thing and act another*” (FC004) meant some people did not follow the rules (#34, +2). The public should have known enough to follow rules that aimed to keep us all safe but such actions by the Government made it less surprising when people did not (#28, +3; #34, +2; #59, -4; #7, -3).

The inept Government response was compounded by an NHS that was not fit for purpose (#10, +6) - “*We just didn’t have enough staff, allied health, nurses, doctors, we didn’t have enough resources. We just didn’t have enough of everything*.” (FC004). The lack of funding for social care services resulted in an overreliance on the NHS (#9, +3). This meant that people were dying from preventable illnesses because our systems were not strong enough to cope with the pandemic (#10, +6; #2, +2).

Despite a lack of trust in the government, there was great trust in the vaccines (#48, +4; #56, -5). Vaccine development brought relief and a way out of the pandemic (#21, +4). However, again the government’s response to vaccine distribution was wrong – the UK should not have been stockpiling vaccines (#43, +3). Besides this being morally wrong, this increased the risk and worry of new variants emerging from countries with low vaccination rates (#11, +2).

It was imperative that the government were held to account for their poor decisions (#40, +5). They should have been doing everything in their power to keep people safe but this was clearly not the case (#26, -3; #44, -2; #45, -1; #25, +5; #32, +5). Their failure to act meant those who were already in vulnerable positions were hit hardest (#4, +4; #60, +3; #51, +2)

## Factor 2 – Fear and anger at policy and public responses

Participants defining Factor 2 were deeply concerned that the portrayal of, and the response to, the virus, both by government and by members of society, led to preventable deaths, unnecessary harms and devastating impacts on people’s mental health (#2, +6; #18, +6; #7, +5; #20, +5; #16, +4; #52, +4; #53, -3; #6, -3).

Government and the media were responsible for creating fear in society (#20, +5; #27, -4; #6, -3; #4, -2; #1, +1) in which “*compliance [was] now a virtue, no matter how ridiculous the request*” (FC022). The response to the pandemic left no room for any alternative views, and the speed at which people fell into line was alarming. Friends, family, neighbours and acquaintances “*became incredibly authoritarian*” (FC024) and began “*asking you to do ridiculous things that didn’t make any sense*” (FC024), such as washing the shopping or using hand sanitiser, and neighbours started to act like “*secret police*” (FC024) (#28, +1).

Decisions such as lockdown were unfair, “*a dangerous road to go down*” (FC067) that caused unnecessary harm, perhaps even more than the virus, particularly to poor inner-city children (#7, +5; #12, +5; #16, +4; #25, -3; #6, -3). Generally, children as a group were not vulnerable to the virus which made closing schools particularly unfair. School closures had a devastating impact on education and mental health for young people, some of who were no longer able to escape from difficult home lives (#18, +6; #7, +5; #52, +4). Staying inside was an easy way for Government to cover for an underfunded NHS, rather than a way to keep people healthy (#55, +4). Yet, and despite such restrictions, people continued to catch COVID-19 from going into hospital with other illnesses and died from preventable illnesses (#2, +6; #12, +5; #51, +2; #10, +1).

Labour and the Conservatives used the pandemic “*as a stick to beat each other with*” (FC067) and the Government did not have “*a bloody clue*” (FC036) about how to deal with the pandemic (#35, -5; #49, +4). There was a lot of misinformation around COVID-19 but as “*some Government guidance was challenged and seemed to be contradictory, you started to question what you were being told*” (FC067) this could have impacted on your mental health and lead you to “*listen to the alternative news*” (#18, +6; #35, -5; #31, -4; #40, +3; #34, +3). Systems to trace those infected with the virus were “*a load of rubbish*” (FC036) and only served to enrich those with connections to the establishment, who benefited from lucrative government contracts, and not help the general public (#14, -4; #35, -5; #26, -3; #34, +3; #40, +3). Government also failed to protect all of their citizens, particularly the self-employed, from the financial effects of the pandemic and for not paying essential workers what they deserved (#13, +3; #26, -3; #37, -2; #51, +2; #36, +2; #17, +2). While more could have been done to stop COVID at its source in China, and by organisations such as the World Health Organisation to stop its spread, particularly in the early days, the Government failed its citizens with poor border control that enabled COVID to enter the country from elsewhere (#46, -5; #26, -3; #44, +2; #45, +1; #50, -1).

The approach to vaccinations is, for factor 2, emblematic of the overall response to the pandemic as what should have been a solution turned out to be more of a problem. Instead of bringing relief to people there was suspicion, worry and mistrust because of the speed and processes of vaccine development and roll-out, pharmaceutical companies being profit driven and concerns of short and long-term side-effects (#33, -6; #48, -5; #21, -4; #56, +4; #58, +3). While vaccines may have been a risk worth taking for those who were vulnerable from COVID-19, *“for those who are not vulnerable, like schoolchildren, it’s insane to give them the vaccine because of two things, it doesn’t protect them against something (…) [and] there’s always a sort of an unknown risk”* (FC060). Personal experience and social media stories of people having bad reactions to the vaccination or even dying from it supported these short-term safety concerns. “*We are all guinea pigs*” (FC048), particularly as vaccines do not make everybody safe (#33, -6; #19, +3; #15, +2; #23, -2). Then when you have had vaccinations it was “*ridiculous*” (FC028) that you needed a vaccine pass to attend large-scale indoor events (#57, -6)

## Factor 3 – Governing through a crisis

For Factor 3 the COVID-19 pandemic was an unprecedented global tragedy but recognised the positive aspects of the pandemic both in terms of the world’s response to it and how it changed our outlook on life for the better (#50, +6; +22, +6; #21, +5; #5, +5; #2, +5; #18, +4; #53, -6).

The COVID-19 pandemic was “*unlike anything in living memory*” (FC063) (#53, -6; #1, -5; #42, -4; #4, -3; #19, +2). It required drastic action, global cooperation and led “…*us, as nations, to have to make unprecedented choices in public policy*” (FC014) (#50, +6; #39, -6; #15, -3). As in other major crises, such as the World Wars, those “*who govern any kind of population [through a crisis] make a hell of a lot of mistakes*” which can result in fatalities (FC052). However, Government were “*trying to do the best for everyone*” (FC015) and should not be held to account for poor decisions as making judgements about what could have been done differently was only possible with “*retrospection*” (FC014) (#26, +4; #40, -4; #37, +3; #12, -2; #46, -2; #25, +1; #51, -1).

Government had clear strategies, “*both politically and clinically*” (FC014), on how to deal with the pandemic and adapt to new learning about the virus, new variants and the population’s behaviour (#35, +3; #37, +3; #47, -2; #31, +1; #32, -1). Tough decisions needed to be made. Responses to the pandemic, such as lockdown, exerted a devastating effect on people’s mental health and led to loneliness, family separation and harms to education (#18, +4; #52, -4; #59, -5; #16, -3; #2, +5; #57, +2). However, these decisions were not unfair, the priority was to reduce deaths and infection (#52, -4 #7, -4; #16, -3; #15, -3). Other countries, such as China, or organisations, like the World Health Organisation were not responsible for the spread of the virus (#44, -4; #45, -2).

The pandemic, while devastating, also highlighted areas of positivity across different spheres of society. The rapid development, and distribution, of safe vaccines was an example of how public and private partnerships can form on a national and global scale to tackle a global threat (#50, +6; #21, +5; #48, +4; #33, +2; #56, -5; #53, -6; #58, -2). Media spread “*disinformation, misinformation*” (FC007), particularly on social media around vaccines (#53, -6; #48, +4; #20, +3; #28, +1). Whereas the reality was that not only have vaccines “*saved more lives globally than any other single healthcare intervention in history”* (FC014) they were a massive relief to many as they gave us “*the ability to see people*” (FC047) again and to have a social life (#21, +5; #23, -1). Making sure everyone around the world was vaccinated helped to keep us all safe from variants (#33, +2; #43, +1; #11, +1). From an individual perspective, the pandemic made us reanalyse how we lived, worked, and interacted with each other for the better (#22, +6; #5, +5).

## Factor 4 – Injustices exposed

Factor 4 paints a picture of the COVID-19 pandemic, unlike anything before it, exposing inequalities at the individual, societal and global levels (#17, +6; #13, +6; #43, +5; #60, +5; #54, +3; #9, +3).

At the individual level, the low-pay of essential workers was a gross injustice which needed to change (#13, +6; #5, +5; #12, +2; #51, +2). Being in lockdown made us realise *“…how much we’re relying on these people getting paid absolutely nothing and running around dropping parcels off so we can continue to live*” (FC032), basically doing “*all the things that we don’t normally think about that just keep everything ticking along*” (FC012). It was unjust that “*we see what you’re doing but not in any way which we’re going to financially recompense you*” (FC008). This was even more pronounced for vulnerable essential workers who had been supported more by family, the community or charities than the government (#13, +6; #12, +2; #51, +2). We proved that we can make changes at short notice when required, we needed to financially compensate essential workers in a way that reflects how much we value them as a society (#13, +6; #5, +5; # 50, +4; #6, +2).

While pretty much everyone was going to catch COVID-19 in the end, the effects of the pandemic were felt disproportionately by the worse-off: “*the richer you are…the better able you were to protect yourself*” (FC008) (#17, +6; #60, +5; #19, +4; #18, +3). There was a wide variety of reasons why this was the case, but the main one was, that unlike some essential workers, you did not need to “*go out and interface with other people in the community*” (FC030). Being richer insulated you and meant you were more likely to use technology to keep your social life and job going online (#17, +6; #41, +3).

At the societal level, existing inequalities meant that people from ethnic minorities suffered more than others in society; the pandemic exposed the deep-rooted racism that exists in society (#60, +5; #54, +3). The Government let down these groups and others with existing vulnerabilities who instead had to rely on support from family, community action or charities (#60, +5; #51, +2; 12, +2). This was positive in the sense that “*people have become more aware of their neighbours, more aware of the local community*”. (# 5, +5; #22, +4; #6, +2; #28, -4).

At the global level, richer nations were able to better protect themselves from the effects of the virus, and rich countries stockpiled COVID-19 vaccinations (#17, +6; #43, +5). Vaccines were the best way to tackle COVID-19 and their development brought a sense of relief (#48, +4; #21, +3; #56, -5; #58, -3) but the “*western world scrambled to get the most vaccines*” (FC006) exposing a lack of care for the whole of society and humanity (#43, +5).

The pandemic was an “*unparalleled*” (FC006) event with devastating impacts on health and mental health (#50, +4; #18, +3; #2, +2; #3, +2; #53, -6; #1, -5; #44, -3; #42, -2; #45, -2). The Government had to act (#39, -6) and there was some sympathy for their response in the sense that “*people want to blame the Government for something which is actually an act of nature*” (FC006). However, the Government had a poor overall strategy for dealing with the pandemic, their communication was poor and restrictions, such as border control and track and trace, were not a success (#46, -5; #35, -4; #14, -4; #26, -2; #40, +1, #34, +1). In contrast, the general public, by and large, behaved well and supported each other (#28, -4; #59, -3; #22, +4; #51, +2). The Government should not force people into following guidelines. Instead, people should, in most areas, have been allowed to use their common sense and get on with things (#23, -4; #27, -3; #11, -1; #57, -1; #19, +4; #3, +2; #15, +1; #8, +1).
