# Supplementary material for: From polarity to plurality: Perceptions of COVID‐19 and policy measures in England and Scotland
Source: Health Expect. 2024 May 11;27(3):e14069. doi: 10.1111/hex.14069 (PMC11087883; doi:10.1111/hex.14069)
Supplement: Supplementary file 3 — Supporting information. [file HEX-27-e14069-s002.docx]

**PARTICIPANT INFORMATION SHEET**

**Perspectives on the COVID-19 Pandemic**

**Introduction and participant information**

You are being invited to take part in a study on ‘Perspectives on the COVID-19 pandemic’, which is being carried out by Glasgow Caledonian University. Before you decide, it is important for you to understand why the study is being conducted and what it will involve. Please take time to read the following information carefully and discuss it with others if you wish. Please do not hesitate to ask the research team questions (see contact details on page three of this document) if there is anything that is unclear or if you would like more information. Take time to decide whether or not you wish to take part.

**Why is this study being carried out?**

COVID-19 has affected everyone in some way. Different people have had different experiences of the virus itself and the range of responses introduced to tackle it. In this project, we are interested in your view on COVID-19. Understanding different perspectives on COVID-19 could help develop approaches for future crises, as well as being able to more effectively support those impacted by the pandemic.

**Why have you been chosen?**

We have invited you to take part in this research because we think you have an important viewpoint and we hope you will share your views with us. We are seeking as broad a range of opinions as possible so we may ask you to recommend others whose views are different to yours.

**Do you have to take part?**

No, participation is voluntary. If you agree to take part in the study, you will complete a consent form. You may withdraw from the study at any point without the need to give a reason (subject to General Data Protection Regulation 2018, the information collected may still be used).

**Who is conducting the research?**

The research is being carried out by a research team at the Yunus Centre for Social Business and Health - Glasgow Caledonian University. The research team includes: Dr Neil McHugh, Dr Jack Rendall and Professor Rachel Baker.

**What will happen if you take part?**

In this study, we use an approach known as Q methodology, which involves asking people to sort cards, printed with statements, onto a grid according to how like their point of view they are. This information is then used to describe in detail the shared views that people have.

The card sort involves sorting cards which contain statements people have said about COVID-19 and the responses to the pandemic. After the card sort we will ask you some questions ‘about you’ to help us describe our sample, further understand your views and to ensure we survey a wide range of people with different characteristics.

A sample of 40-60 individuals will do the card sort exercises. Each card sort, interview and ‘about you’ questions will take approx. 60 minutes.

We are collecting data in three different ways: virtually; by post; or face-to-face. You can choose to take part in whatever way suits you best. **Virtually:** the card-sort, interview and ‘about you’ questions will take place online with one of the researchers at a pre-arranged time. This requires you to have access to a computer i.e. a laptop or a desktop. **By-post**: the card-sort will be posted out to you with instructions for you to self-complete. One of the researchers will conduct the interview and the ‘about you’ questions via a video or phone call. **Face-to-face:** the card-sort, interview and ‘about you’ questions will be conducted in person on the GCU campus, your place of work or in a suitable public space. The latest Government guidance will be followed for all face-to-face interactions.

You will be asked to provide consent confirming your agreement to take part in the research study prior to data collection.

**Will you benefit directly from this research study?**

There is no benefit to you personally in taking part. However, your experiences and views will be included in our findings which will be used to inform policy.

**What will happen to the information that you give?**

All information provided will be confidential. Only members of the research team will have access to it. Your name will not be used in any report or publication produced. In accordance with the University’s policy on data protection (and under General Data Protection Regulation 2018), your data will not be kept longer than 5 years after project completion. Anonymised personal data will be kept secure and protected by a password on a GCU computer. Anonymous research data will be deposited in the [UK Data Service Repository](https://ukdataservice.ac.uk/). If you have any questions about the University’s data protection policy, you can talk to a member of the research team (details below) or send an email to dataprotection@gcu.ac.uk.

**Do I have to take part?**

It is up to you to decide whether or not to take part. Even if you decide to take part, you can withdraw at any time, without giving a reason.

**Your rights**

This study is being conducted on the lawful basis of ‘public interest’ (set out in Article 6(1)(e) and Article 9(2)(j) of General Data Production Regulation 2018 and the UK Data Protection Act 2018). Subject to this legislation, you have the right to be informed; you may also have the right to access, rectify, erase or restrict your data, or object to it being used. If you are dissatisfied with how your information has been handled and the response from the University, you have the right to lodge a complaint with the Information Commissioner's Office (ICO): Address: Information Commissioner's Office, Wycliffe House, Water Lane, Wilmslow, Cheshire SK95AF; T: 0303 123 1113; Email: [casework@ico.org.uk](mailto:casework@ico.org.uk).

**What happens when the research study ends?**

The data will be analysed, and findings will be available through written reports, established website reports, the media, presentations and journal publications. Written reports of the study findings will be shared with the participants upon request.

**Ethics**

This study has been approved by the Ethics Committee, Glasgow School for Business and Society, Glasgow Caledonian University.

**Funding**

This research is part of the *Financial lives and wellbeing in low-income groups post Covid-19 (FinWell-Covid)* project, funded by the Economic and Social Research Council (ESRC).

**Further information**

If you would like any further information about this study, please contact:

Jack Rendall

Email: [jack.rendall@gcu.ac.uk](mailto:jack.rendall@gcu.ac.uk)

Neil McHugh

Email: [neil.mchugh@gcu.ac.u](mailto:neil.mchugh@gcu.ac.u)k

Rachel Baker

Email: [rachel.baker@gcu.ac.u](mailto:rachel.baker@gcu.ac.u)k

Yunus Centre for Social Business and Health

M201, 2nd Floor, George Moore Building,

Glasgow Caledonian University

Cowcaddens Road

Glasgow G4 OBA
