# Supplementary material for: From polarity to plurality: Perceptions of COVID‐19 and policy measures in England and Scotland
Source: Health Expect. 2024 May 11;27(3):e14069. doi: 10.1111/hex.14069 (PMC11087883; doi:10.1111/hex.14069)
Supplement: Supplementary file 4 — Supporting information. [file HEX-27-e14069-s001.docx]

**CONSENT FORM**

**Perspectives on the COVID-19 Pandemic**

This form asks you to give consent to take part in this research project. Please read the form carefully and ensure that you understand all parts of it. If you require assistance with this form, please inform the researcher using the contact details provided below.

| • I have read and understood the information sheet for the above study. |
| --- |
| • I have had the opportunity to ask questions. |
| • I have received satisfactory answers to all my questions. |
| • I understand that my participation is voluntary, and I am free to withdraw from the study at any time and without having to give a reason, without any legal rights being affected. |
| • I understand that my data will not be shared with the government or third-party organisations. |
| • I understand results and individual quotes may be published; however, it will not be possible to identify me in future publications. |
| • I understand that anonymous research data will be deposited in a responsible digital repository (the UK Data Service Repository). |
|  |
| • I am an adult and 18 years or older. |
| • I freely consent to take part in this study. |
| • I am willing to have the interview audio recorded. |

If you agree to all parts of the form, please send a reply to the email below confirming your agreement and participation in the research. If you have any further questions, please contact one of the researchers and they will be happy to answer your queries.

Researcher contact details:

Jack Rendall. E: [jack.rendall@gcu.ac.uk](mailto:jack.rendall@gcu.ac.uk)

Neil McHugh. E: [neil.mchugh@gcu.ac.uk](mailto:neil.mchugh@gcu.ac.uk)
